# Clonidine-stimulated growth hormone concentrations (cut-off values) measured by immunochemiluminescent assay (ICMA) in children and adolescents with short stature

**DOI:** 10.6061/clinics/2016(04)09

**Published:** 2016-04

**Authors:** Maria de Fátima Borges, Flávia Carolina Cândida Teixeira, Aline Karin Feltrin, Karina Alvarenga Ribeiro, Gabriel Antonio Nogueira Nascentes, Elisabete Aparecida Mantovani Rodrigues Resende, Beatriz Pires Ferreira, Adriana Paula Silva, Heloísa Marcelina Cunha Palhares

**Affiliations:** I Universidade Federal do Triângulo Mineiro (UFTM), Divisão de Endocrinologia, Unidade de Endocrinologia Pediátrica, Uberaba/MG, Brazil; II Instituto Federal do Triângulo Mineiro (IFTM), Microbiologia e Imunologia, Uberaba/MG, Brazil

**Keywords:** Clonidine, GH Deficiency, Short Stature, Idiopathic Short Stature

## Abstract

**OBJECTIVES:**

To establish cut-off values for growth hormone concentrations using clonidine as a secretagogue and an immunochemiluminescent assay as the method of measurement and to analyze the response time as well as the influence of gender, nutritional status and pubertal stage.

**METHODS:**

A total of 225 tests were performed in 3 patient groups, categorized as group 1 (normal), group 2 (idiopathic short stature) and group 3 (growth hormone deficiency). Among the 199 disease-free individuals, 138 were prepubertal, and 61 were pubertal. Clonidine (0.1 mg/m^2^) was orally administered, and the growth hormone level was measured by immunochemiluminescent assay. The growth hormone peak and the difference between the growth hormone peak and the baseline level were then analyzed. Statistical analyses were performed using Student's t-test or the Mann-Whitney test and Kruskal-Wallis test followed by Dunn's post hoc test. Cut-off values were determined using a receiver operating characteristic curve.

**RESULTS:**

Group 1 and group 2 had no difference in growth hormone peak, gender, body mass index standard deviation score, or pubertal stage. Group 3 exhibited a significantly lower growth hormone peak than the other groups did. The receiver operating characteristic curve demonstrated that growth hormone concentrations ≥ 3.0 ng/mL defined responsiveness to clonidine. In total, 3.02% of individuals in group 1 and group 2 were considered false positive, i.e., these children lacked growth hormone deficiency and had a peak below 3.0 ng/mL.

**CONCLUSION:**

Clonidine-stimulated growth hormone concentrations ≥3 ng/mL, as measured by immunochemiluminescent assay, suggest responsiveness to the stimulus regardless of gender, body mass index standard deviation score or pubertal stage.

## INTRODUCTION

After birth, growth in height results from endogenous factors, such as hormones and genetic background, and from exogenous factors, such as proper nutrition and the psychosocial and emotional conditions under which the individual develops 1,2.

Growth rate and weight gain are the main parameters used by pediatricians to monitor the height development and health of a child. When short stature or a growth rate decrease is detected, the child should be evaluated according to an investigative protocol for short stature to define the etiology and appropriate therapy 3.

In specific protocols, children with certain conditions diagnosed during the evaluation of short stature, such as growth hormone (GH) deficiency (GHD) as well as Turner syndrome (TS) in girls in particular, can receive recombinant human GH (rhGH) therapy from the Brazilian Ministry of Health (Ministério da Saúde do Brasil). However, for this, it is first necessary to perform a differential diagnosis between these conditions and familial, constitutional and/or idiopathic short stature (ISS) in addition to other diseases that manifest with short stature 4.

GHD can be detected directly, by measurements of GH and its effector, insulin-like growth factor-1 (IGF-1), or indirectly, by evaluation of the growth rate over a year. Under normal conditions, basal GH concentrations are low and often undetectable. The evaluation of GH secretion can be performed by determining either the integrated GH secretion over 12 to 24 hours, which is an unfeasible methodology from a clinical point of view, or the GH concentration after pharmacological stimulation, with a lack of response to at least two different secretagogues indicating a problem 5. The most frequently used pharmacological stimuli in Brazil are clonidine, an α-agonist drug that promotes the release of GH-releasing hormone (GHRH) 6, and insulin-induced hypoglycemia 7, in addition to glucagon, arginine, L-dopa and exercise, among others 5.

It has been arbitrarily agreed that the plasma-stimulated GH level (the GH peak) that is indicative of adequate secretion is ≥10 ng/mL, as assayed by radioimmunoassay (RIA) 6-11. Below this cut-off, individuals are considered as GH deficient within the appropriate clinical context 2,3.

Beginning in the 1990s, more sensitive and specific immunometric methods were developed in addition to international benchmarks for measuring GH and other hormones, such as luteinizing hormone (LH), thyroid-stimulating hormone (TSH), and IGF-1. New reference standards for measurements of the baseline and cut-offs in stimulation tests were then imposed, suggesting the need to adapt treatment protocols 12-15.

Since 1996, concentrations of GH have been determined at the Federal University of Triangulo Mineiro (Universidade Federal do Triângulo Mineiro - UFTM) by immunochemiluminescent assay (ICMA), and clonidine and insulin are the most commonly used GH secretagogues. In the present study, to reassess the reference standards and to determine the cut-off value for the GH peak, 225 clonidine stimulation tests performed between 2000 and 2014 were evaluated. Other features, such as the time to the greatest response, gender differences and the influence of prepubertal and pubertal developmental stages, were also studied.

## SUBJECTS AND METHODS

This prospective study was approved by the UFTM Ethics Committee. Patients were selected in the Pediatric Endocrinology Unit, where they were evaluated for short stature according to the standardized protocol for conduct in the clinic. All patients completed an initial form covering their history, and a physical examination was performed. The following anthropometric data were also calculated: height standard deviation score (SDS), growth velocity SDS, weight SDS, body mass index (BMI) and BMI SDS 16. Additionally, the pubertal stage was determined 17,18, and the pertinent data were plotted on growth charts for Brazilian children 19. After general biochemical tests, TSH testing, x-rays of the skull and bone age determination according to Greulich and Pyle 20 were performed, quarterly monitoring of the patients was initiated. The first stimulation test was requested after monitoring the growth rate for a time ranging from 6 to 12 months, unless panhypopituitarism or central nervous system (CNS) tumors had been immediately identified. The conditions that resulted in the test being indicated for a specific child were short stature, a height SDS <-2, a decreased growth velocity according to age and a tendency toward a shift in the growth path observed in the growth chart 3,5.

When the test was indicated, by the morning (7:00 am), the child was greeted in the functional testing room by a physician and a nurse trained to conduct the exam. The child was then placed in the supine position, and a vein was punctured for infusion of 0.9% saline. After 40 minutes of rest, the first 5 mL sample of whole blood was collected. The GH concentration in this sample was considered as the baseline and was used for comparison with the stimulated concentrations. After obtaining the baseline sample, 0.10 mg/m^2^ clonidine was administered orally, and then blood samples were collected after 30, 60, 90 and 120 minutes. The whole blood was centrifuged at 4000 rpm, and the serum was then separated and kept at -20°C until the hormone assay.

GH concentrations were determined by ICMA using an automated system (IMMULITE 1000Immunoassay System, Siemens, Berlin, Germany) and commercial kits obtained from DPC (Diagnostic Products Corporation, Los Angeles, CA, USA). This sandwich assay uses two antibodies specific for the circulating 22 kDa form of GH and the standard calibrator 80/505 from the World Health Organization, with a detection limit of 0.02 µg/L (1 µ/L = 1 ng/mL) and an intra-assay coefficient of 5.7% for GH concentrations of 0.02 to 5 µg/L 21.

In a previous pilot study using the same method, as subsequently confirmed by other authors 5,22, increases in clonidine-stimulated GH concentrations ≥5 ng/mL were considered as a normal response, and all of the diagnostic and clinical decisions considered this figure as a cut-off value.

For cases where there was a lack of response to the clonidine test, the second test was the insulin tolerance test (ITT), used according to conventional descriptions 7,11, and a GH concentration ≥5 ng/mL was assumed to indicate a normal response. None of these tests was preceded by testosterone or estradiol administration to sensitize the test (priming). The GH peak (the greatest increase achieved) and ΔGH (the difference between the greatest increase and the baseline) were considered for all of the samples obtained during each of the tests.

In addition to the GH stimulation tests, girls with short stature had their karyotypes determined according to the GTG-banding technique, with an analysis of 100 metaphases 23. IGF-1 concentrations were not determined in the laboratory of the UFTM, but rather were outsourced to other laboratories and thus were determined according to variable technical and reference standards, which made correlation with the GH response to secretagogues unfeasible 15.

For evaluation of the clonidine test, the selected cases were divided into 3 groups:

- Group 1 (G1) consisted of 56 children without disease and with small delays in weight and a height SDS >-2. They were later considered as the control group because the longitudinal follow-up demonstrated that these children did not have disease and that in most cases, their heights normalized. This group was categorized based on the height SDS calculated during the consultation during which the GH stimulation test with clonidine was indicated. The indication for the test was justified by a reduction in the growth rate or by a temporary shift in growth.
- Group 2 (G2) consisted of 143 patients with ISS and a height SDS <-2. Children who were small for gestational age (SGA) or who had chronic clinical conditions were not included.
- Group 3 (G3) consisted of 26 children with GHD. Seven cases were due to panhypopituitarism, 13 children had isolated deficiency characterized by decreased growth velocity (<2 cm/year) that improved with rhGH therapy, and 6 children had craniopharyngioma 3,5.

### Statistical analysis

The data distribution was analyzed using the Kolmogorov-Smirnov test, and the homogeneity of variances was verified by Levene's test. As our data showed a nonparametric distribution, for comparisons of two independent samples (including comparisons between males and females and between the prepubertal and the pubertal stages within each clinical group), the Mann-Whitney test was used, and for comparisons of three independent groups, the Kruskal-Wallis test followed by Dunn's post hoc test was used. Correlations between the GH peak and the BMI SDS were obtained using the Spearman test.

Finally, a receiver operating characteristic (ROC) curve was constructed by calculating the sensitivity and specificity for all possible cut-off values of the GH peak. The area under the ROC curve represents the probability that the test will distinguish affected individuals from unaffected individuals. For the best cut-off value in each comparison group, the sensitivity with a 95% confidence interval (CI), the specificity (95% CI) and positive and negative predictive values (95% CI) were obtained.

Comparisons and data from the ROC curve analysis were obtained using SPSS version 20 and MedCalc 10.3 software, respectively. The observed differences were considered significant when the significance level (*p*) was less than 0.05.

## RESULTS

A total of 225 GH stimulation tests were analyzed, with 199 of them performed in children categorized as normal based on the height SDS (G1; n=56) or as having ISS (G2; n=143). Additionally, 26 tests were performed in children with GHD (G3).

G1 (height SDS ranging from normal to ≥-2) consisted of 56 children, including 33 boys and 23 girls. These children were aged 10.4 years.months (yr.mo) (median) (minimum: 3.6 and maximum: 14.0), with a height SDS of -1.53 (-1.94 to 1.96) and a BMI SDS of -0.59 (-1.80 to 1.10). The median baseline GH concentration was 0.27 ng/mL (0.05 to 3.00). The maximum value in response to the test (GH peak) was 8.00 ng/mL (2.00 to 23.00). These data as well as bone age and ΔGH values are shown in table 1.

In this group, 6 (10. 71%) patients were considered nonresponsive to the clonidine test according to the reference standards at the time (≥5 ng/mL); these patients underwent the ITT, in which they exhibited normal responses (GH peak ≥5 ng/mL), suggesting that they had been false positive for GHD when subjected to the clonidine test.

G2 (height SDS <-2) consisted of 143 children, including 83 boys and 60 girls, aged 9.0 yr.mo (median) (minimum: 3.0 and maximum: 14.7). These children had a height SDS of -2.70 (-5.24 to -2.30) and a BMI SDS of -0.80 (-1.60 to 2.0). The median baseline GH concentration was 0.37 ng/mL (0.01 to 2.30), and the GH peak response was 9.00 ng/mL (1.00 to 36.00). These data as well as bone age and ΔGH values are shown in Table 1. In this group, 21 (14.68%) patients were considered nonresponsive to the clonidine test, underwent the ITT and yielded a normal response.

In G3, 26 children had a proven diagnosis and outcome of GHD. The age of these children was 9.3 yr.mo (minimum: 3.11 and maximum: 15.4), and they had a height SDS of -3.60 (-9.44 to -2.16) and a BMI SDS of -0.18 (-4.50 to 3.00). The median baseline GH concentration was 0.33 ng/mL (0.05 to 2.29), and the GH peak response was 1.00 ng/mL (0.00 to 2.20). These data as well as bone age and ΔGH values are shown in Table 1. All patients in this group underwent the ITT and were nonresponsive in this test as well as the previous test. In the ITT, the baseline GH concentration was 0.33 ng/mL (0.10 to 0.59), and the GH peak was 0.40 ng/mL (0.05 to 1.00).

### Intra- and inter-group comparisons

Comparisons between the three groups are depicted in table 1. As expected, when all of the groups were compared, the height SDS showed a significant difference (G2,G3<G1; G3<G1,G2) between the groups, and the bone age in G2 was lower than that in G1. There was no significant difference in the baseline GH, but the GH peak concentrations (as well as ΔGH) in G3 were lower than those in G1 and G2. No differences were found between G1 and G2 (Table 1).

To study the influence of gender and puberty on the GH response to clonidine, the patients from G1 and G2 were divided into male, female, prepubertal and pubertal subgroups; in this last subgroup, the patients were in Tanner stages 2 and 3. Except for differences in chronological age and certain expected significant differences in anthropometric data, there were no significant differences in the baseline or stimulated GH level in G1 (Table 2) and G2 (Table 3) according to gender or pubertal development.

In these groups, there was also no correlation between the GH peak (as well as ΔGH) and the BMI SDS, independent of gender or puberty (*p*>0.005). The greatest GH increases occurred after 60 minutes in 63.31% of the subjects, after 90 minutes in 25.62% and after 120 minutes in 6.03%.

**Figure 1:**
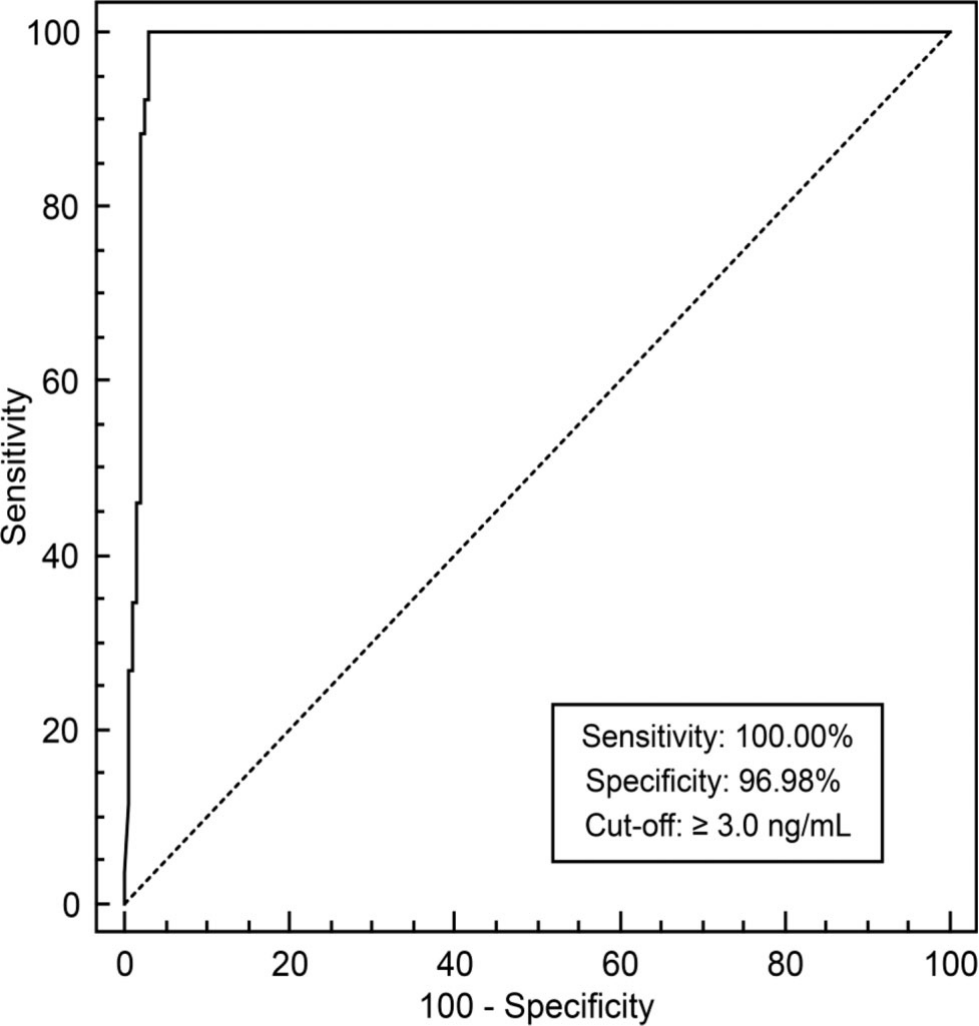
ROC curve showing a value of 3.0 ng/mL for stimulated GH (GH peak) as the reference value (cut-off), which was able to differentiate normal children (G1) and children with ISS (G2) from children with GHD (G3).

Construction of the ROC curve initially aimed to establish whether one group could be differentiated from another based on the GH peak and ΔGH parameters. G1 compared with G2 proved to be unable to define a possible growth deficit according to the GH evaluation. Combined G1+G2 and G2 were then tested against G3, and both comparisons were significant (*p*<0.001), i.e., both the groups of children with ISS (G2) and the normal children could be differentiated from the children with GHD by the ROC curve. The best parameter for differentiating the groups was the GH peak, and a 3.0 ng/mL value was the cut-off. In this case, the GH peak demonstrated a sensitivity of 100.00% (86.80% to 100.00%) and a specificity of 96.98% (93.60% to 98.90%) in identifying children with GHD (Figure 1).

## DISCUSSION

Studies conducted in past decades have presented data supporting the therapeutic use of rhGH in children with isolated GHD or GHD in association with another deficiency (panhypopituitarism) as well as in children with ISS or who are SGA; in children with chronic renal failure; and in children with genetic syndromes such as TS, Noonan syndrome and Prader-Willi syndrome 24. Although rhGH therapy is temporary for most of these conditions, it must be permanent in cases of GHD; therefore, a differential diagnosis should be performed with the other conditions, particularly in children with ISS or who are SGA 3,5,24.

Children with GHD are identified when they do not respond to two GH stimulation tests, and despite all of the controversies involving the need for these GH tests 25,26, the tests still provide important data for differential diagnosis relative to other conditions that can present a clinical context similar to that of isolated or idiopathic GHD 2,3.

In certain states in Brazil, including the state of Minas Gerais, rhGH is provided only to children with GHD or TS. Furthermore, in children with GHD, rhGH is only provided when there is a lack of a GH response to two provocative tests, even when there is a relevant clinical context 4,5.

As soon as the immunoradiometric, immunofluorometric (IFMA) and ICMA methods were developed, experience working with them demonstrated that a cut-off value of 10 ng/mL for the GH response to provocative tests was too high for both the clonidine test and the ITT, leading to several false-positive diagnoses 12-15. In a pilot study 22 involving 78 patients, a cut-off value more appropriate for the ICMA method was adopted by our service (5 ng/mL), thus providing a foundation for the current study.

Certain authors have suggested steroid priming for GH stimulation tests, using initial administration of testosterone or estradiol to achieve the traditional response of 10 ng/mL, as with an RIA 27. We consider this artifice unnecessary because the real goal is to differentiate children with GHD from normal children or children with ISS, and to achieve this goal, a very high cut-off level that has been arbitrarily defined in a less sensitive and less specific method is not necessary 12-15.

In the current study, side effects of the clonidine administered during the GH stimulation tests were observed in 23% of the patients, as reported in the literature 28. However, only somnolence and mild hypotension, with no need for oral hydration or saline infusion, were observed, as suggested by several authors 28,29. Batista et al. 30 showed that a low dose of clonidine (0.1 mg/m^2^) is effective as a GH-stimulating agent, resulting in lesser side effects. Since then, we have standardized this clonidine dose, and our data agree with those authors' findings. Klein et al. 31 studied the pharmacokinetics and pharmacodynamics of the most employed clonidine dose, namely, 0.15/m^2^, when administered to 40 children. The researchers found a higher serum concentration than necessary to trigger GH release according to model-based predictions, and they proposed that a lower dose would be sufficient as a clonidine challenge and could result in milder side effects.

In the current study, children were categorized based on their height SDS to create a clinical spectrum that included normal children and those with ISS (G2). We did not find any differences in gender, developmental stage (pubertal or prepubertal) or the GH response to clonidine between G1 and G2. Furthermore, the children were followed in the long term during use of simple measures, such as a healthier diet and exercise, and they spontaneously recovered their growth paths based on their family characteristics and according to normal curves, which clinically validated their laboratory data.

It is currently known that spontaneous GH secretion increases during puberty, in parallel with increasing concentrations of sexual steroids 32. However, most reports on GH-provoking tests were performed in the prepubertal period 6,9,10,12,13,27,30. In contrast, studies comparing GH peak concentrations in response to secretagogues between the prepubertal and the pubertal periods are rare 26, so this aspect is still controversial. In the present study, we did not find any difference in respect to this issue, similar to the findings of Cavallo et al. 32 and Ghigo et al. 11. These authors observed a GH response to several release-inducing agents, including oral clonidine, and similarly to the present report, their pubertal patients were in Tanner stages 2 and 3. Whether the inclusion of patients in more advanced stages of puberty, with certainly higher steroid concentrations, could have changed our results is a question that remains to be answered.

Comparing the other two groups with the GHD group, GH peak concentrations and ΔGH were significantly lower in the GHD group. In addition, the ROC curve revealed that a cut-off value of 3.0 ng/mL was able to differentiate patients with GHD from those in the other groups.

The cut-off value of 3.0 ng/mL found in the present study was similar to that in a previous study in which the authors used immunometric methods 12; this is the minimal GH response that a patient must reach to be considered responsive to the clonidine test without the need for priming. Using this cut-off value to analyze the patients from G1 and G2, only 6 (3.02%) patients would need the second test (ITT), whereas the cut-off value of 5 ng/mL resulted in 27 (13.56%) patients needing to undergo the ITT.

Demonstrating and promoting a criterion for a positive response to the clonidine test with a lower cut-off value in the context of short stature, the Brazilian Unified Health System (SUS) would have justification to not provide rhGH for these children. However, as stated previously, the protocols need to be adapted and to accept all GH indications supported by the medical literature, which goes well beyond providing GH only for patients with GHD or TS. The other causes of short stature may require rhGH temporarily, which will help the individual to achieve a stature that is compatible with good quality of life and equal opportunities.

Certain authors have suggested that obesity can influence the GH response to clonidine stimulation 34. In the current study, individualizing the weight and BMI of each child according to age and gender by calculating the BMI SDS, there was no difference between the groups, and no correlation with the GH peak was observed; therefore, our data do not support this proposition.

As observed by Galluzzi et al. 35, 93.96% of stimulated GH peaks occurred within 90 minutes in G1 and G2. Therefore, the testing period could be standardized to 90 minutes, as adopted in the European and American protocols, which would reduce the discomfort and cost of the procedure 30.

Based on the data obtained in the current study, we conclude that the clonidine-stimulated GH concentrations ≥3.0 ng/mL that were assayed by ICMA, within an appropriate clinical and laboratorial context, indicated normal GH responsiveness to stimulation. This value is independent of gender, BMI SDS and pubertal developmental stage. Moreover, the clonidine test could be performed with 3 blood collections, at baseline, 60 minutes and 90 minutes, to improve the patient's comfort and reduce the costs.

## AUTHOR CONTRIBUTIONS

Borges MF developed the project, coordinated the work and wrote the manuscript. Feltrin AK, Teixeira FC and Ribeiro KA assisted the patient selection according to the methods reported in this work and selected and plotted the data in Excel and in the Tables. Resende EA, Ferreira BP and Palhares HM guided the selection of the patients according to the methods reported in this work, the performance of the GH stimulation tests and the careful treatment and follow-up of the patients. Nascentes GA and Silva AP performed the statistical analysis and configured the tables and figure. Palhares HM helped in writing the manuscript.

## Figures and Tables

**Table 1:** Anthropometric data from and baseline and clonidine-stimulated (0.10 mg/m^2^ BS) GH concentrations in normal subjects (G1), in patients with ISS (G2), and in patients with GHD (G3), with the ITT performed in nonresponsive clonidine patients.

DATA	G1 (n: 56)	G2 (n: 143)	G3 (n: 26)	Between groups^1^ Comparisons *p*-value
Age^2^ (yr.mo)	10.4 (3.6→14.0)9	9.0 (3.0→14.7)	9.3 (3.11→15.4)	0.344
Bone Age (yr.mo)	9.0 (2.0→13.0)	7.0 (1.0→14.0)	5.0 (2.0→13.0)	0.031(G1>G2)
Height SDS^3^	-1.53 (-1.94→1.96)	-2.70 (-5.24→-2.30)	-3.60 (-9.44→-2.16)	0.0001(G2<G1; G3<G1; G3<G2)
GV SDS^4^	-2.00 (-6.00→0.00)	-2.00 (-8.00→0.00)	-4.00 (-7.00→-1.00)	0.0003(G3<G2; G3<G1)
BMI^5^ SDS	-0.59 (-1.80→1.10)	-0.80 (-1.60→-2.00)	-0.18 (-4.50→3.00)	0.131
Baseline GH (ng/mL)	0.27 (0.05→3.00)	0.37 (0.01→2.30)	0.33 (0.05→2.29)	0.187
GH Peak^6^ (ng/mL)	8.00 (2.00→23.00)	9.00 (1.00→36.30)	1.00 (0.00→2.20)	< 0.0001(G3<G1; G3<G2)
ΔGH^7^	7.60 (0.30→22.65)	8.87 (0.00→33.00)	0.00 (0.00→2.00)	< 0.0001(G3<G1; G3<G2)
ITT^8^ GH Peak (ng/mL)	12.00 (5.50→21.00)	11.70 (6.00→23.30)	0.40 (0.05→1.00)	-

1: Kruskal-Wallis test followed by Dunn's test;

2: Chronological age represented in years (yr.) and months (mo.);

3: SDS = standard deviation score;

4: GV SDS = growth velocity SDS;

5: BMI = body mass index;

6: GH Peak = maximum clonidine-stimulated GH concentration;

7: ΔGH = GH peak – baseline GH

8: G1 (10.71%; n=6) and G2 (14.68%; n=21);

9: Values expressed as the median (minimum and maximum = minimum → maximum).

**Table 2:** Anthropometric data from and baseline and clonidine-stimulated GH concentrations in normal patients (G1), distributed and compared according to gender and pubertal development.

	Male (n=33)			Female (n=23)			Male *vs* Female	
Group 1	Prepubertal (n=23)	Pubertal (n=10)	*p*-value^1^	Prepubertal (n=12)	Pubertal (n=11)	*p*-value^1^	Prepubertal *p*-value^1^	Pubertal *p*-value^1^	
Age^2^ (yr.mo)	10.90 (4.90→13.70)7	12.80 (10.60→14.00)	< 0.0001	8.50 (3.60→11.10)	11.20 (9.11→13.00)	< 0.0001	< 0.0001	0.014
Bone Age (yr.mo)	9.00 (3.00→13.00)	12.50 (8.00→13.00)	< 0.0001	8.00 (2.00→12.00)	9.00 (7.00→13.00)	< 0.0001	0.562	0.227
Height SDS^3^	-1.15 (-1.94→1.96)	-1.60 (-1.91→-1.24)	0.946	-1.88 (-1.93→108)	-1.57 (-1.92→0.09)	0.003	0.014	0.706
BMI^4^ SDS	-0.61 (-1.40→1.00)	-1.85 (-1.80→-0.20)	0.027	-0.52 (-1.70→1.10)	-0.15 (-1.10→0.90)	0.194	0.377	0.004
Baseline GH (ng/mL)	0.30 (0.05→1.90)	0.10 (0.05→2.10)	0.497	0.20 (0.05→3.00)	0.71 (0.05→2.00)	0.190	0.439	0.234
GH Peak5 (ng/mL)	9.00 (3.00→23.00)	9.00 (3.00→22.00)	0.653	8.00 (2.00→17.00)	8.00 (3.00→14.00)	0.311	0.472	0.442
ΔGH^6^ (ng/mL)	8.92 (1.15→22.65)	8.78 (3.27→22.00)	0.572	7.16 (0.30→14.80)	7.21 (2.87→13.42)	0.156	0.305	0.435

1: Mann-Whitney test;

2: Chronological age represented in years (yr.) and months (mo.); 3: SDS = standard deviation score;

4: BMI = body mass index;

5: GH Peak = maximum clonidine-stimulated GH concentration;

6: ΔGH = GH peak – baseline GH.

7: Values expressed as median (minimum and maximum = minimum→maximum).

**Table 3:** Anthropometric data from and baseline and clonidine-stimulated GH concentrations in patients with ISS (G2), distributed and compared according to gender and pubertal development.

	Male (n=83)			Female (n=60)			Male *vs* Female	
Group 2	Prepubertal (n=62)	Pubertal (n=21)	*p*-value^1^	Prepubertal (n=41)	Pubertal (n=19)	*p*-value^1^	Prepubertal *p*-value^1^	Pubertal *p*-value^1^
Age^2^ (yr.mo)	8.20 (3.00→14.70)7	13.70 (10.00→14.60)	< 0.0001	6.60 (3.40→13.90)	11.50 (9.01→13.50)	< 0.0001	0.035	< 0.0001
Bone Age (yr.mo)	4.20 (1.00→14.00)	11.00 (6.70→14.00)	< 0.0001	4.20 (1.00→11.00)	10.00 (6.00→13.00)	< 0.0001	0.240	0.117
Height SDS^3^	-2.81 (-5.24→-2.30)	-2.78 (-5.05→-2.30)	0.704	-2.67 (-5.20→-2.25)	-2.35 (-3.54→-2.10)	0.045	0.692	0.189
BMI^4^ SDS	-0.83 (-1.60→1.70)	-1.42 (-1.30→-0.60)	0.020	-0.52 (-1.20→1.70)	-0.64 (-1.20→0.80)	0.106	0.031	0.135
Baseline GH (ng/mL)	0.44 (0.01→2.30)	0.40 (0.05→1.80)	0.591	0.47 (0.01→2.29)	0.34 (0.08→1.87)	0.788	0.636	0.654
GH Peak^5^ (ng/mL)	8.78(0.65→33.30)	8.70 (3.30→25.00)	0.518	8.40 (0.00→36.30)	9.45 (3.50→19.80)	0.614	0.887	0.934
ΔGH^6^ (ng/mL)	8.00 (0.00→33.00)	7.00 (1.00→25.00)	0.361	7.00 (0.00→33.00)	9.00 (2.00→19.00)	0.488	0.544	0.727

1: Mann-Whitney test;

2: Chronological age represented in years (yr.) and months (mo.);

3: SDS = standard deviation score;

4: BMI = body mass index;

5: GH Peak = maximum clonidine-stimulated GH concentration;

6: ΔGH = GH peak – baseline GH

7: Values expressed as the median (minimum and maximum = minimum → maximum).

## References

[1] Styne D, GardnerShoback D G D (2007). Growth. Greenspan's Basic & Clinical Endocrinology.

[2] Hoffman DJ (2014). Growth retardation and metabolic programming: implications and consequences for adult health and disease risk. J Pediatr (Rio J).

[3] Cohen P, Rogol AD, Deal CL, Saenger P, Reiter EO, Ross JL (2008). Consensus statement on the diagnosis and treatment of children with idiopathic short stature: a summary of the Growth Hormone Research Society, the Lawson Wilkins Pediatric Endocrine Society, and the European Society for Paediatric Endocrinology Workshop. J Clin Endocrinol Metab.

[4] Ministério da Saúde, Gabinete do Ministro: Portaria n.° 110, de 10 de março de 2010 (2010). Protocolo cl&iacute;nico e diretrizes terap&ecirc;uticas - Defici&ecirc;ncia de horm&ocirc;nio do crescimento &ndash; hipopituitarismo [Clinical protocol and therapeutic guidelines - Growth hormone deficiency - hypopituitarism]. Brasília, Diário Oficial da União, DF.

[5] De Paula PL, Czepielewski MA.Evaluating diagnosis methods for childhood GH (DGH) deficiency: IGFs, IGFBPs, releasing tests, GH rhythm and image exams (2008). Arq Bras Endocrinol Metabol.

[6] Gil-Ad I, Leibowitch N, Josefesberg Z, Wasserman M, Laron Z (1990). Effect of oral clonidine, insulin- induced hypoglycemia and exercise on plasma GHRH levels in short-stature children. Acta Endocrinol (Copenh).

[7] Kaplan SL, Abrams CA, Bell JJ, Conte FA, Grumbach MM (1968). Growth and growth hormone. I. Changes in serum level of growth hormone following hypoglycemia in 134 children with growth retardation. Pediatr Res.

[8] Gil-Ad I, Topper E, Laron Z (1979). Oral clonidine as a growth hormone stimulation test. Lancet.

[9] Fraser NC, Seth J, Brown NS (1983). Clonidine is a better test for growth hormone deficiency than insulin hypoglycemia. Arch Dis Child.

[10] Loche S, Cappa M, Chigo E, Faedda A, Lampis A, Carta D (1993). Growth hormone response to oral clonidine test in normal and short children. J Endocrinol Invest.

[11] Chigo E, Bellone J, Aimaretti G, Bellone S, Loche S, Cappa M (1996). Reliability of provocative test to assess growth hormone secretory status. Study in 472 normally growing children. J Clin Endocrinol Metab.

[12] Silva EGP, Slhessarenko N, Arnhold IJP, Batista MC, Estefan V, Osorio MGF (2003). GH values after clonidine stimulation measured by immunofluorometric assay in normal prepubertal children and GH-deficient patients. Horm Res.

[13] Chaler EA, Rivarola MA, Guerci B, Ciaccio M, Costanzo M, Travaglino P (2006). Differences in serum GH CUT-off values for pharmacological testes of GH secretion depend on the serum GH method. Clinical validation from the growth rate score during the first year of treatment. Horm Res.

[14] Tenenbaum-Rakover Y (2008). The need to revise the cut-off level for the diagnosis of GH deficiency in children. Pediatr Endocrinol Rev.

[15] Clemmons DR (2011). Consensus statement on the standardization and evaluation of growth hormone and insulin-like growth factor assays. Clin Chem.

[16] Tanner JM, Whitehouse RH, Takaishi M (1966). Standards from birth to maturity for height, weight, height velocity, and weight velocity: british children, 1965. Part II. Arch Dis Child.

[17] Marshal WA, Tanner JM (1969). Variations in the pattern of pubertal changes in girls. Arch Dis Child.

[18] Marshal WA, Tanner JM (1970). Variations in the pattern of pubertal changes in boys. Arch Dis Child.

[19] Marques RM, Marcondes E, Berquó E, Brand R, Yunes J (1982). Crescimento e Desenvolvimento Pubert&aacute;rio em Crian&ccedil;as e Adolescentes Brasileiros. Altura e Peso [Growth and Pubertal Development in Brazilian Children and Adolescents. Height and Weight].

[20] Greulich WW, Pyle SI (1959). Radiographic Atlas of Skeletal Development of the Hand and Wrist, 2^nd^ ed.

[21] L'Hermite-Balériaux, Copinschi G, Van Cauter E (1996). Growth hormone assays: early to latest test generations compared. Clin Chem.

[22] Borges MF, Dalalio V, Brito VS, Resende EM, Ferreira BP, Mauro KM (1999). Avalia&ccedil;&atilde;o do teste de est&iacute;mulo do hGH com clonidina utilizando o m&eacute;todo de quimioluminesc&ecirc;ncia automatizado [Evaluation of the hGH stimulation test with clonidine using an automated chemiluminescence method]. Arq Bras Endocrinol Metabol.

[23] Scheres JM (1972). Human chromosome banding. Lancet.

[24] Cook DM, Rose SR (2012). A review of guidelines for use of growth hormone in pediatric and transition patients. Pituitary.

[25] Cianfarani S, Tondinelli T, Spadoni GL, Scire G, Boemi S, Boscherini B (2002). Height velocity and IGF-I assessment in the diagnosis of childhood onset GH insufficiency: do we still need a second GH stimulation test. Clin Endocrinol (Oxf).

[26] Gandrud LM, Wilson DM (2004). Is growth hormone stimulation testing in children still appropriate. Growth Horm IGF Res.

[27] Martínez AS, Domené HM, Ropelatto MG, Jasper HG, Pennisi PA, Escobar ME (2000). Estrogen priming effect on growth hormone (GH) provocative test: a useful tool for the diagnosis of GH deficiency. J Clin Endocrinol Metab.

[28] Marui S, Oliveira CHMC, Souza SCAL, Berger K, Khawali C, Hauache OM (2015). Tolerance of the oral clonidine test in 180 patients: efficacy of saline resuscitation in controlling arterial hypotension. Arq Bras Endocrinol Metab.

[29] May M, Rose SR (2007). Oral hydration during growth hormone stimulation with clonidine. Old IJJ Pediatr Nurs.

[30] Batista MC, Arnhold IJ, Mendonça BB, Abronzo FH, Bloise W, Nicolau W (1987). Low-dose oral clonidine: effective growth hormone releasing agent in children but not in adolescents. J Pediatr.

[31] Klein RH, Alvarez-Jimenez R, Sukhai RN, Oostdijk W, Bakker B, Reeser HM (2013). Pharmacokinetics and pharmacodynamics of orally administered clonidine: a model-based approach. Horm Res Paediatr.

[32] Rose SR, Municchi G, Barnes KM, Kamp GA, Uriarte MM, Ross JL (1991). Spontaneous growth hormone secretion increases during puberty in normal girls and boys. J Clin Endocrinol Metab.

[33] Cavallo L, Acquafredda A, Liuzzi S, Russo R, Zecchino C, Leuzzi R (1992). Growth hormone release during insulin tolerance, clonidine, arginine and growth hormone releasing hormone tests in short normal children and adolescents. J Endocrionol Invest.

[34] Loche S, Guzzetti C, Pilia S, Ibba A, Civolani P, Porcu M (2011). Effect of body mass index on the growth hormone response to clonidine stimulation testing in children with short stature. Clin Endocrinol (Oxf).

[35] Galluzzi F, Stagi S, Parpagnoli M, Losi S, Pagnini I, Favelli F (2006). Oral clonidine provocative test in the diagnosis of growth hormone deficiency in childhood: should we make the timing uniform. Horm Res.

